# Disease burden and prediction of liver cancer attributable to metabolic risks in five East Asian countries from 1990 to 2023

**DOI:** 10.1371/journal.pone.0342628

**Published:** 2026-08-07

**Authors:** Jialu Wang, Huijiao Wang, Zixuan Huang, Xiling Liu, Dehua Wang, Huimin Yan

**Affiliations:** 1 School of Public Health, Hebei Medical University, Shijiazhuang, China; 2 Hebei Key Laboratory of Immune Mechanism of Major Infectious Diseases and New Technology of Diagnosis and Treatment, The Fifth Hospital of Shijiazhuang, Shijiazhuang, China; Public Library of Science, UNITED STATES OF AMERICA

## Abstract

Liver cancer is a major public health issue in five East Asian countries, with metabolic factors becoming primary drivers. Based on the Global Burden of Disease (GBD) 2023 database, we extracted the total number and age-standardized mortality rates, years lived with disability (YLDs), years of life lost (YLLs) and disability-adjusted life years (DALYs) related to liver cancer attributable to metabolic risks (LC-MR). We analyzed the disease burden of LC-MR and trends across five East Asian countries from 1990 to 2023, examining patterns by sex and age as well as key drivers across different nations, and projected future trends through 2053. In 2023, Mongolia exhibited the highest age-standardized rates for LC-MR, while China ranked first in the total absolute number of cases. From 1990 to 2023, the overall disease burden showed a downward trend in Japan and Republic of Korea, whereas an upward trend was observed in Mongolia, Democratic People’s Republic of Korea, and China. Significant sex and age disparities were observed across all countries in that males bore a higher burden and the burden generally shifted towards the elderly population. Decomposition analysis revealed that the changes in disease burden in Japan and Republic of Korea were primarily driven by population ageing, whereas the main drivering factors were epidemiological changes and population growth in Mongolia and Democratic People’s Republic of Korea. Projections of disease burden for 2053 indicate that the disparities among countries would further widen. Therefore, strengthening metabolic risk interventions and implementing targeted prevention strategies for high-risk populations, especially the aging population, is crucial for reducing the regional burden of LC-MR, given the risk disparities across different countries.

## Introduction

Liver cancer is a serious global public health concern. According to GLOBOCAN 2022 statistics, the global incidence rate of liver cancer ranks sixth and the mortality rate ranks third among malignant tumors [1]. Due to the hidden symptoms and limitations in early diagnostic techniques, the majority of patients of liver cancer are diagnosed at an advanced stage, with the five-year survival rate ranging from 5% to 30%. This brings substantial challenges to global disease management programs and imposes a severe financial and health burden on patients and their families [2].

Hepatitis B virus (HBV) and hepatitis C virus (HCV) infections have long been the primary causative factors for liver cancer. However, the etiological composition of liver cancer worldwide has undergone substantial shifts in recent years. With rising obesity rates and lifestyle changes, metabolic risk factors represented by obesity, type 2 diabetes mellitus (T2DM), and metabolic dysfunction-associated steatohepatitis (MASH) have become the fastest-growing contributors to liver cancer incidence [3]. A cohort study confirms a significant positive correlation between these metabolic risk factors and both liver cancer incidence and mortality [4]. Furthermore, co-exposure to multiple metabolic risk factors exerts synergistic effects, further elevating disease risk. This trend also suggests that these factors play a crucial role in elucidating the underlying mechanisms of liver cancer. However, despite progress in elucidating the pathogenesis of liver cancer, many key mechanisms remain unclear. Among these, metabolic reprogramming in hepatocellular carcinoma (HCC), a critical feature driving its progression, exhibits complex associations with multiple metabolic factors. This reprogramming involves extensive remodeling of metabolic pathways for carbohydrates, lipids, and amino acids, among which many alterations, such as the Warburg effect and enhanced lipid synthesis, are closely associated with MASH development [5]. Meanwhile, abnormal glutamine metabolism exacerbates insulin resistance, thereby increasing the risk of T2DM [6]. Moreover, obesity and metabolic disorders in HCC further form a mutually reinforcing vicious cycle. These mechanistic associations further confirm the central role of metabolic risk factors in the initiation and progression of liver cancer.

Notably, Asia accounts for over 75% of all incidences of liver cancer worldwide, and the epidemiological trend of metabolic-associated liver disease (MAFLD) in the region is a major concern. The incidence of MAFLD increased by 11.2% globally between 2010 and 2021, with East Asia exhibiting the largest growth (16.6%), greatly exceeding that of other areas [7]. The five East Asian countries (China, Japan, Republic of Korea, Democratic People’s Republic of Korea, and Mongolia) share relatively similar population genetic backgrounds while exhibiting socioeconomic development gradients. This spectrum includes the low-income closed economy of Democratic People’s Republic of Korea, the fast-developing huge economy of China, the middle-income resource-based country of Mongolia, and the high-income developed economies of Republic of Korea and Japan. Furthermore, the region as a whole is undergoing a change in the spectrum of illnesses from infectious to metabolic liver disorders. In particular, the prevalence of HBV-, HCV-related viral liver diseases has decreased significantly across countries with the widespread use of hepatitis B vaccination and antiviral agents. In contrast, the disease burden of Metabolic Dysfunction-Associated Steatotic Liver Disease (MASLD) has increased dramatically, becoming the most common liver illness. The prevalence of MASLD reached 37% in China [8], the incidence in South Korea increased from 10.49% in 2012 to 17.13% in 2022 [9], and the proportion of cirrhosis associated with non-alcoholic steatohepatitis in Japan rose from 2.0% to 9.1% [10]. This transition poses new challenges to public health systems, necessitating enhanced early screening and multidisciplinary management.

Although the impacts of metabolism-related liver cancer have become increasingly pronounced in the five countries, current relevant research has several limitations. First, there is still a lack of regional coverage. Previous studies have focused on China, Japan, and South Korea, but lack complete data on North Korea and Mongolia, making it difficult to fully represent the general features of the five East Asian countries [11]. Second, causality analysis is still undetermined. Since 1990, there have been few systematic assessments of the isolated contribution of metabolic hazards to long-term trends. Most current studies focus on the overall burden of liver cancer or specific metabolic variables. This makes it impossible to measure their relative importance in the development of causality.

From 1990 to 2023, this study aimed to systematically analyze the mortality rate, years of life lost (YLL), years lived with disability (YLD), and disability-adjusted life years (DALYs) of liver cancer attributable to metabolic risks (LC-MR). Standardized methods of statistics were used, based on the Global Burden of Disease (GBD) 2023 database, to explore the disease burden’s periodic development trend, age and sex specificity, and geographical heterogeneity. Additionally, this study explored the relationship and dynamic change trend between the disease burden and the Socio-demographic Index (SDI) in five East Asian countries. Decomposition analysis was used to determine the contribution of significant factors, such as aging, population growth, and epidemiological shifts, to the variation in disease burden. Finally, the future development trend of the disease burden was predicted using the Bayesian Age-Period-Cohort (BAPC) model. In addition to optimizing the distribution of public health resources and reducing the regional burden of liver cancer, this study will serve as a guide for countries and regions around the world with comparable disease spectrum and metabolic risk evolution characteristics in order to develop focused prevention and control strategies.

## Methods

### Data source and country selection

The Global Burden of Disease (GBD) 2023 database integrates data from civil registration, verbal autopsy, surveys, censuses, surveillance systems, and cancer registries, and estimates age‑standardized mortality rates, disability‑adjusted life years (DALYs), and other burden metrics for 204 countries and territories, covering 375 diseases and injuries and 88 risk factors [12]. The data for this study were sourced from the GBD 2023 database, retrieved using the GHDx-Results Tool on October 15, 2025. No individual-level identifiable information was accessible to the authors throughout the study period, as the GBD database provides only de-identified aggregate data. The time range selected was 1990–2023. The indicators chosen to measure disease burden were Deaths, DALYs, YLDs, and YLLs. The selected geographic region comprised five East Asian countries: China, Democratic People’s Republic of Korea, Japan, Mongolia, and Republic of Korea. The GBD estimate selected was “Risk factor”, the risk category selected was “Metabolic risks”, and the cause selected was “Liver Cancer” (GBD cause code: B.1.7). Liver cancer was defined according to the International Classification of Diseases, Ninth Revision (ICD-9) codes 155.0–155.1 and Tenth Revision (ICD-10) codes C22.0–C22.9. In the GBD 2023 framework, estimates of disease burden attributable to risk factors are generated using the comparative risk assessment framework independently of the primary aetiological classification of liver cancer. Therefore, the attributable burden of metabolic risks reflects the proportion of overall liver cancer burden estimated by GBD to be associated with metabolic risk exposures at the population level. Furthermore, the GBD 2023 study analyzed the disease burden stratified by quintiles of the SDI. The SDI is a composite indicator that encompasses per capita income, average years of schooling, and the fertility rate among females under the age of 25 in each geographic unit. SDI scores range from 0 to 100, with higher scores indicating better socioeconomic conditions. Population data were based on the GBD World Standard Population.

### GBD modelling strategy and burden estimation

The GBD study employed advanced methods to estimate disease metrics for liver cancer. These estimates were derived using DisMod-MR 2.1 (Disease Modelling-Meta Regression Tool Version 2.1), which integrates literature, hospital, and claims data and incorporates healthcare accessibility and quality indices as key covariates. Cause-specific estimates involved independent modeling for specific causes, using relationships among prevalence, cause-specific mortality rates, and excess mortality rates. These relationships were modeled using MR-BRT (Meta-Regression-Bayesian Regression, Regularisation, Trimming). Detailed modeling strategies are outlined in the official GBD methodology appendix (https://www.healthdata.org/gbd/methods-appendices-2021). DALY denotes the total loss of healthy life years attributable to disease, focusing on the aggregate burden of disease, and is defined by equation (1). YLD represents health loss due to disability, focusing on quality of life, and is calculated using equation (2). YLL denotes the loss of life years due to premature death, focusing on survival time, and is calculated using equation (3).


$$\begin{array}{c} \text{DALY }=\text{ YLL }+\text{ YLD} \end{array}$$ (1)

$$\begin{array}{c} \text{YLD }=\text{ prevalence }\times \text{ disability weight} \end{array}$$ (2)

$$\begin{array}{c} \text{YLL }=\text{ number of deaths }\times \text{ standard life expectancy} \end{array}$$ (3)

### Statistical analysis

#### Expected annual percentage change (EAPC).

To quantify the overall long-term trends, we calculated the EAPC by fitting a linear regression to the natural logarithm of annual rates. Years were coded sequentially as *t* = 0, 1, 2, …, 33 (1990 as the index year). The regression model was specified as equation (4), where Rt represents the rate in year *t*, *α* denotes the intercept, *β* is the regression coefficient, and *ε* is the error term.


$$\begin{array}{c} \text{ln}(Rt)\;=\;\alpha \;+\;\beta t\;+\;\varepsilon \end{array}$$ (4)

The EAPC was calculated using equation (5), with 95% confidence interval (CI) derived from the standard error of *β*. The trend was considered statistically significant if the confidence interval excluded zero.


$$\begin{array}{c} \text{EAPC }=\;\left(\hat{e}\beta \;-\;\text{1}\right)\;\times \;\text{1}\text{0}\text{0}\% \end{array}$$ (5)

### Spearman’s rank correlation analysis

Spearman’s rank correlation coefficient (*rho*) was calculated to assess the relationship between the SDI and the rates of deaths, DALYs, YLDs, and YLLs. The method for calculating Spearman’s correlation coefficient *rho* is shown in equation (6). For each pair of variables, the observed values were converted to ranks. Let *xᵢ* and *yᵢ* denote the ranks of the i-th observation for the two variables, and let *x̄* and *ȳ* be their respective means. In this analysis, we considered the correlation to be weak if the absolute value of *rho* fell between 0.1 and 0.3, moderate if between 0.3 and 0.5, and strong if the *rho* was greater than 0.5.


$$\begin{array}{c} ho\;=\;\frac{\Sigma \left[\left(x_{i}\;-\;\bar{x}\right)\left(y_{i}\;-\;\bar{y}\right)\right]}{\sqrt{\left[\Sigma {\left(x_{i}\;-\;\bar{x}\right)}^{\text{2}}\;\Sigma {\left(y_{i}\;-\;\bar{y}\right)}^{\text{2}}\right]}} \end{array}$$ (6)

### Decomposition analysis

To quantify the drivers of changes in disease burden, we conducted a decomposition analysis to assess the independent contributions of ageing, population growth, and epidemiological shifts [13]. This study employs the Das Gupta decomposition method to break down changes in disease burden between 1990 and 2023 into contributions from these factors.

### Bayesian age-period-cohort model

The Bayesian age-period-cohort (BAPC) model, which is crucial for capturing dynamic epidemiological aspects [14], has been widely validated and applied in studies involving age-structured population data and complex cohort effects [15]. In this study, the BAPC model was fitted using the BAPC package in R, which implements Bayesian inference through the INLA framework. The model was applied to age-specific disease burden rates, and the projected age-standardized rates were subsequently calculated based on the predicted age-specific estimates. Future disease burden trends were projected through 2053.

### Analytical software

This study employed R statistical software (version 4.4.3) for data analysis and visualization. Specific analyses leveraged R packages including “ggplot2”, “dplyr”, “RcolorBrewer”, and “patchwork”. A two-tailed *P-value* less than 0.05 was considered statistically significant.

### Ethics statement

The dataset utilised in this study was derived entirely from the GBD 2023 database. The implementation of this database adhered to the ethical guidelines outlined in the Declaration of Helsinki and complied with regulatory requirements in relevant jurisdictions. As this secondary analysis employed only publicly accessible aggregated data from open-source epidemiological databases, ethics committee approval was not required under applicable institutional guidelines.

## Results

### The disease burden and trends of LC-MR from 1990 to 2023

**Fig 1:**
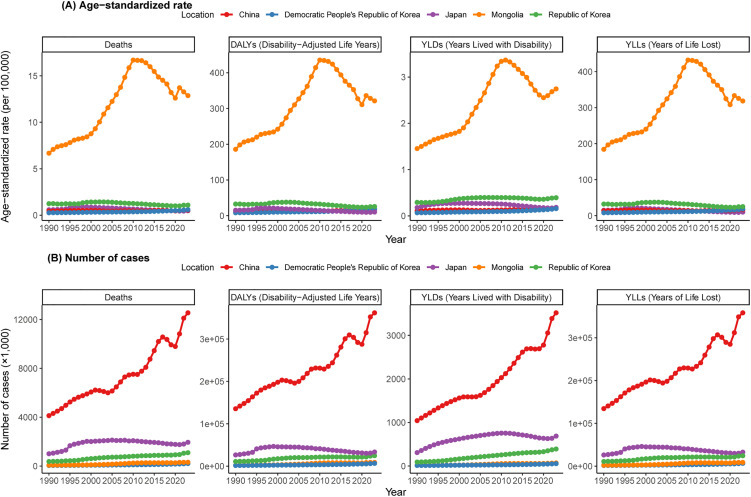
Trends from 1990 to 2023 in number and age-standardized rates for LC-MR in five East Asian countries. (A) Age-standardized rate; (B) Number of cases.

In 2023, among the five East Asian countries, Mongolia’s age-standardized mortality rate for LC-MR was 12.87 per 100,000 person-years, whereas this indicator was significantly lower and relatively similar across the other countries, ranging from 0.48 to 1.09 per 100,000 person-years. A similar pattern was observed in the standardized rates of DALYs, YLDs and YLLs (Table 1, Fig 1A). However, China recorded the highest numbers of mortality, DALYs, YLDs, and YLLs, with 12,551, 361,862, 3,520, and 358,343 cases, respectively (Table 1, Fig 1B).

**Table 1:** Changing patterns of LC-MR in five East Asian countries in 1990 and 2023.

Sex	Metric	location	1990 Number (95% UI)	1990 ASR (95% UI)	2023 Number (95% UI)	2023 ASR (95% UI)	EAPC (95% CI)
Both	DALYs	China	135566 (82995 to 215129)	13.79 (8.57 to 21.68)	361862 (204675 to 544826)	16.61 (9.28 to 25.05)	0.03 (-0.17 to 0.23)
		Democratic People’s Republic of Korea	1558 (665 to 3101)	8.22 (3.59 to 16.37)	6560 (2601 to 12808)	18.68 (7.43 to 36.18)	2.18 (2.02 to 2.33)
		Japan	26497 (15126 to 41844)	15.32 (8.73 to 24.14)	33437 (17808 to 53585)	10.24 (5.31 to 17.27)	-2.2 (-2.7 to -1.69)
		Mongolia	2105 (951 to 3855)	185.83 (84.57 to 340.7)	8949 (4270 to 14698)	321.38 (155.42 to 510.5)	2.14 (1.57 to 2.72)
		Republic of Korea	11079 (5584 to 18449)	32.66 (17.11 to 52.91)	24735 (13353 to 37691)	25.67 (13.85 to 39.63)	-1.08 (-1.43 to -0.73)
	Deaths	China	4129 (2609 to 6411)	0.45 (0.29 to 0.68)	12551 (7463 to 18483)	0.56 (0.33 to 0.81)	0.23 (0.04 to 0.41)
		Democratic People’s Republic of Korea	46 (20 to 92)	0.27 (0.12 to 0.51)	208 (88 to 400)	0.6 (0.26 to 1.15)	2.17 (2 to 2.33)
		Japan	1009 (584 to 1624)	0.59 (0.34 to 0.94)	1949 (1042 to 3172)	0.48 (0.26 to 0.77)	-1.56 (-2.11 to -1.01)
		Mongolia	72 (34 to 130)	6.68 (3.18 to 12.08)	313 (152 to 496)	12.87 (6.5 to 20.43)	2.61 (2.04 to 3.19)
		Republic of Korea	375 (201 to 597)	1.25 (0.71 to 1.95)	1095 (610 to 1631)	1.09 (0.61 to 1.63)	-0.7 (-0.99 to -0.41)
	YLDs	China	1043 (564 to 1694)	0.11 (0.06 to 0.18)	3520 (1605 to 5715)	0.16 (0.07 to 0.26)	0.64 (0.46 to 0.81)
		Democratic People’s Republic of Korea	12 (5 to 26)	0.07 (0.03 to 0.14)	55 (20 to 118)	0.16 (0.06 to 0.34)	2.29 (2.13 to 2.45)
		Japan	309 (147 to 518)	0.18 (0.09 to 0.3)	688 (314 to 1242)	0.18 (0.09 to 0.33)	-0.87 (-1.35 to -0.39)
		Mongolia	16 (7 to 32)	1.45 (0.61 to 2.88)	71 (32 to 131)	2.74 (1.26 to 4.88)	2.35 (1.8 to 2.89)
		Republic of Korea	92 (45 to 160)	0.29 (0.15 to 0.5)	392 (188 to 718)	0.39 (0.19 to 0.72)	0.81 (0.53 to 1.09)
	YLLs	China	134523 (82416 to 213615)	13.68 (8.51 to 21.52)	358343 (202543 to 539530)	16.45 (9.18 to 24.83)	0.03 (-0.17 to 0.22)
		Democratic People’s Republic of Korea	1546 (659 to 3074)	8.15 (3.55 to 16.23)	6505 (2579 to 12687)	18.52 (7.37 to 35.83)	2.18 (2.02 to 2.33)
		Japan	26188 (14924 to 41350)	15.14 (8.61 to 23.85)	32749 (17457 to 52600)	10.05 (5.21 to 16.87)	-2.22 (-2.72 to -1.71)
		Mongolia	2089 (943 to 3827)	184.38 (83.81 to 337.67)	8878 (4236 to 14568)	318.63 (154.35 to 505.57)	2.14 (1.57 to 2.71)
		Republic of Korea	10986 (5534 to 18313)	32.37 (16.96 to 52.54)	24344 (13190 to 36883)	25.28 (13.69 to 38.82)	-1.1 (-1.46 to -0.75)
Female	DALYs	China	58847 (38379 to 91156)	12.35 (8.07 to 19.12)	106069 (62882 to 156058)	9.03 (5.32 to 13.33)	-1.56 (-1.77 to -1.35)
		Democratic People’s Republic of Korea	516 (230 to 1102)	5 (2.24 to 10.6)	2831 (1145 to 5889)	14.72 (5.96 to 30.52)	2.93 (2.76 to 3.1)
		Japan	8264 (4713 to 13234)	8.55 (4.87 to 13.69)	12345 (6642 to 19748)	5.86 (3.24 to 9.19)	-2.31 (-2.95 to -1.67)
		Mongolia	924 (359 to 1730)	153.26 (60.35 to 281.09)	3919 (1874 to 6118)	264.66 (125.91 to 412.31)	2.41 (1.73 to 3.09)
		Republic of Korea	3399 (1639 to 5604)	19.19 (9.4 to 31.33)	7335 (4525 to 11243)	13.14 (7.96 to 20.18)	-1.6 (-2 to -1.21)
	Deaths	China	1911 (1242 to 2969)	0.42 (0.27 to 0.65)	4380 (2523 to 6383)	0.36 (0.21 to 0.53)	-0.95 (-1.16 to -0.75)
		Democratic People’s Republic of Korea	18 (8 to 39)	0.19 (0.09 to 0.4)	104 (43 to 213)	0.54 (0.22 to 1.1)	2.79 (2.62 to 2.97)
		Japan	372 (212 to 609)	0.38 (0.21 to 0.61)	829 (434 to 1412)	0.31 (0.17 to 0.49)	-1.68 (-2.36 to -1)
		Mongolia	31 (13 to 57)	5.4 (2.16 to 9.72)	151 (72 to 237)	11.52 (5.56 to 18.13)	3.23 (2.53 to 3.93)
		Republic of Korea	137 (67 to 221)	0.84 (0.41 to 1.34)	410 (252 to 618)	0.69 (0.43 to 1.04)	-0.97 (-1.29 to -0.66)
	YLDs	China	475 (265 to 776)	0.1 (0.06 to 0.17)	1169 (585 to 1839)	0.1 (0.05 to 0.15)	-0.66 (-0.87 to -0.45)
		Democratic People’s Republic of Korea	5 (2 to 11)	0.05 (0.02 to 0.11)	27 (10 to 61)	0.14 (0.05 to 0.31)	3 (2.82 to 3.18)
		Japan	116 (58 to 198)	0.12 (0.06 to 0.2)	300 (140 to 537)	0.13 (0.06 to 0.22)	-0.92 (-1.49 to -0.34)
		Mongolia	7 (3 to 14)	1.19 (0.49 to 2.4)	38 (17 to 65)	2.66 (1.21 to 4.63)	2.84 (2.21 to 3.47)
		Republic of Korea	33 (15 to 60)	0.19 (0.09 to 0.34)	150 (79 to 278)	0.26 (0.13 to 0.47)	0.7 (0.38 to 1.02)
	YLLs	China	58372 (38145 to 90395)	12.25 (8.02 to 18.95)	104899 (62149 to 154622)	8.94 (5.26 to 13.21)	-1.57 (-1.78 to -1.35)
		Democratic People’s Republic of Korea	511 (228 to 1092)	4.96 (2.22 to 10.49)	2804 (1131 to 5826)	14.58 (5.89 to 30.2)	2.93 (2.76 to 3.1)
		Japan	8148 (4664 to 13056)	8.43 (4.82 to 13.5)	12045 (6451 to 19225)	5.74 (3.18 to 8.93)	-2.34 (-2.98 to -1.69)
		Mongolia	917 (356 to 1716)	152.07 (59.89 to 278.82)	3881 (1853 to 6063)	261.99 (124.37 to 407.73)	2.41 (1.73 to 3.09)
		Republic of Korea	3366 (1620 to 5550)	18.99 (9.31 to 31.01)	7185 (4420 to 11009)	12.88 (7.83 to 19.67)	-1.64 (-2.03 to -1.24)
Male	DALYs	China	76719 (41817 to 127402)	15.03 (8.29 to 25.03)	255794 (137408 to 397003)	23.96 (12.76 to 37.22)	0.93 (0.73 to 1.12)
		Democratic People’s Republic of Korea	1042 (399 to 2279)	11.79 (4.76 to 25)	3729 (1149 to 8006)	22.21 (7.02 to 48.13)	1.64 (1.48 to 1.79)
		Japan	18233 (10074 to 28416)	22.68 (12.57 to 35.49)	21092 (10583 to 35501)	14.94 (7.26 to 26.23)	-2.15 (-2.58 to -1.71)
		Mongolia	1181 (483 to 2446)	223.35 (92.23 to 456.37)	5031 (2332 to 8624)	386.12 (183.75 to 657.47)	1.89 (1.42 to 2.36)
		Republic of Korea	7680 (3293 to 13855)	48.52 (21.68 to 86.16)	17400 (9047 to 27115)	38.58 (20.21 to 60.17)	-0.98 (-1.31 to -0.65)
	Deaths	China	2218 (1223 to 3681)	0.47 (0.26 to 0.77)	8170 (4612 to 12654)	0.75 (0.42 to 1.14)	1.03 (0.85 to 1.21)
		Democratic People’s Republic of Korea	28 (11 to 59)	0.35 (0.14 to 0.73)	104 (33 to 227)	0.64 (0.21 to 1.42)	1.55 (1.39 to 1.72)
		Japan	637 (356 to 1018)	0.82 (0.46 to 1.32)	1120 (584 to 1824)	0.68 (0.34 to 1.13)	-1.47 (-1.94 to -1)
		Mongolia	40 (17 to 81)	8.2 (3.45 to 16.01)	162 (77 to 277)	14.29 (6.78 to 24.45)	1.96 (1.52 to 2.4)
		Republic of Korea	238 (105 to 423)	1.79 (0.83 to 3.23)	685 (369 to 1056)	1.54 (0.85 to 2.36)	-0.66 (-0.93 to -0.4)
	YLDs	China	569 (260 to 927)	0.12 (0.05 to 0.19)	2350 (1068 to 3956)	0.22 (0.1 to 0.36)	1.47 (1.32 to 1.63)
		Democratic People’s Republic of Korea	7 (2 to 17)	0.09 (0.03 to 0.2)	28 (9 to 65)	0.17 (0.05 to 0.39)	1.66 (1.51 to 1.81)
		Japan	193 (91 to 330)	0.24 (0.12 to 0.41)	388 (176 to 703)	0.25 (0.11 to 0.43)	-0.84 (-1.25 to -0.43)
		Mongolia	9 (3 to 19)	1.76 (0.63 to 3.64)	33 (14 to 67)	2.78 (1.18 to 5.35)	1.86 (1.39 to 2.32)
		Republic of Korea	59 (25 to 117)	0.41 (0.18 to 0.79)	241 (108 to 422)	0.54 (0.24 to 0.95)	0.76 (0.51 to 1.01)
	YLLs	China	76151 (41557 to 126508)	14.91 (8.23 to 24.85)	253444 (135840 to 393153)	23.75 (12.59 to 36.9)	0.92 (0.72 to 1.12)
		Democratic People’s Republic of Korea	1035 (396 to 2263)	11.71 (4.72 to 24.8)	3701 (1138 to 7943)	22.04 (6.95 to 47.74)	1.64 (1.48 to 1.79)
		Japan	18040 (9950 to 28110)	22.44 (12.41 to 35.06)	20704 (10368 to 34903)	14.7 (7.15 to 25.68)	-2.16 (-2.6 to -1.73)
		Mongolia	1172 (480 to 2427)	221.59 (91.52 to 453.06)	4997 (2313 to 8554)	383.33 (182.14 to 652.83)	1.89 (1.42 to 2.36)
		Republic of Korea	7620 (3270 to 13769)	48.11 (21.51 to 85.51)	17159 (8931 to 26719)	38.05 (19.91 to 59.22)	-1 (-1.33 to -0.67)

Age-standardized rates of death, DALYs, YLDs and YLLs were shown as rates (95% uncertainty interval) per 100,000 person-years. ASR, Age-standardized rate. DALYs, disability-adjusted life years. YLDs, disability adjusted life years. YLLs, years of life lost.

From 1990 to 2023, age-standardized mortality rates showed an increasing trend in Mongolia, Democratic People’s Republic of Korea and China, with Mongolia experiencing the fastest rise (EAPC = 2.61: 95% CI 2.04 to 3.19), whereas Japan and Republic of Korea showed a downward trend, with Japan exhibiting the most pronounced decline (EAPC = −1.56: 95% CI −2.11 to −1.01). The trend for DALYs rates was similar, increasing in Democratic People’s Republic of Korea, Mongolia and China, with both Democratic People’s Republic of Korea and Mongolia exhibiting relatively rapid and comparable rates of increase, whereas Japan and Republic of Korea showed a downward trend, with Japan again showing the most pronounced decline (EAPC = −2.20: 95% CI −2.70 to −1.69). For YLDs rates, all four countries showed an upward trend except for Japan, which declined; Mongolia recorded the fastest rate of increase (EAPC = 2.35: 95% CI 1.80 to 2.89). The trend in the YLL rate was entirely consistent with that of the DALY rate (Table 1, Fig 1).

### Burden of LC-MR by sex

**Fig 2:**
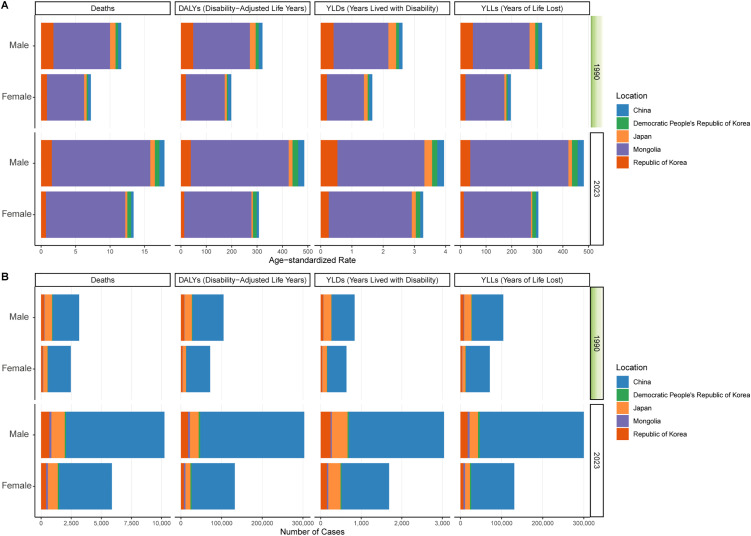
Age-standardized rates of LC-MR by sex in five East Asian countries in 1990 and 2023. (A) Age-standardized Rate; (B) Number of Cases.

Among males in 2023, Mongolia showed by far the highest age-standardized mortality rate for LC-MR, whereas the other countries presented much lower and relatively similar rates, ranging between 0.64 and 1.54 per 100,000 person-years. The rates of DALYs, YLDs and YLLs followed the same pattern (Fig 2B). Regarding trends from 1990 to 2023, age-standardized mortality rates showed an upward trend in Mongolia, Democratic People’s Republic of Korea and China, with Mongolia experiencing the fastest rise (EAPC = 1.96: 95% CI 1.52 to 2.40), whereas they showed a downward trend in Japan and Republic of Korea, with the decline being most pronounced in Japan (EAPC = −1.47: 95% CI −1.94 to −1.00). The trends in DALYs and YLL rates were entirely consistent with those in mortality rates. As for YLD rates, all four countries except Japan showed an increase, with Mongolia exhibiting the fastest rise (EAPC = 1.86: 95% CI 1.39 to 2.32) (Fig 2B).

Among females in 2023, Mongolia showed by far the highest age-standardized mortality rate for LC-MR, whereas the other countries presented much lower and relatively similar rates, ranging between 0.31 and 0.69 per 100,000 person-years. The rates of DALYs, YLDs and YLLs followed the same pattern (Fig 2B). Regarding trends from 1990 to 2023, age-standardized mortality rates showed an upward trend in Mongolia and the Democratic People’s Republic of Korea, with Mongolia experiencing the fastest rise (EAPC = 3.23: 95% CI 2.53 to 3.93), whereas Japan, Republic of Korea and China showed a downward trend, with Japan exhibiting the most pronounced decline (EAPC = −1.68: 95% CI −2.36 to −1.00). DALY, YLL, and YLD rates followed broadly similar directional trends, though the fastest increases were in Democratic People’s Republic of Korea; additionally, YLD rates increased in Republic of Korea (Fig 2B).

### Burden of LC-MR by age group

**Fig 3:**
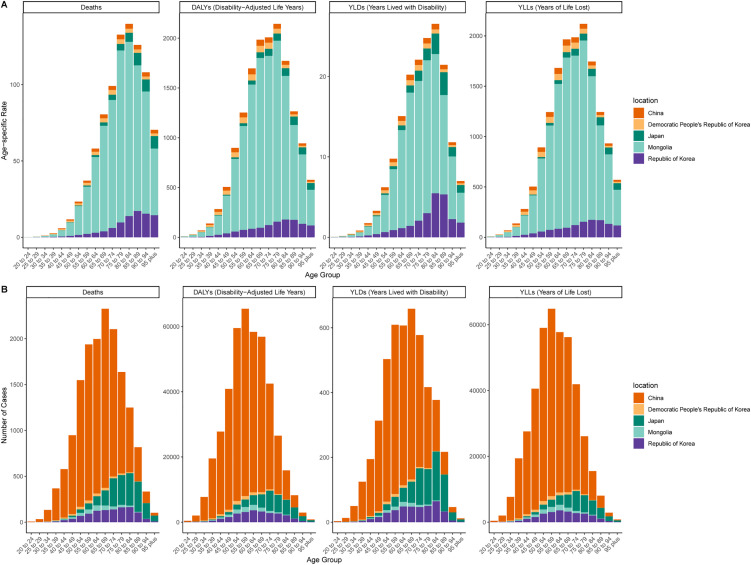
Age-standardized rates of LC-MR by age group in five East Asian countries in 1990 and 2023.

**Fig 4:**
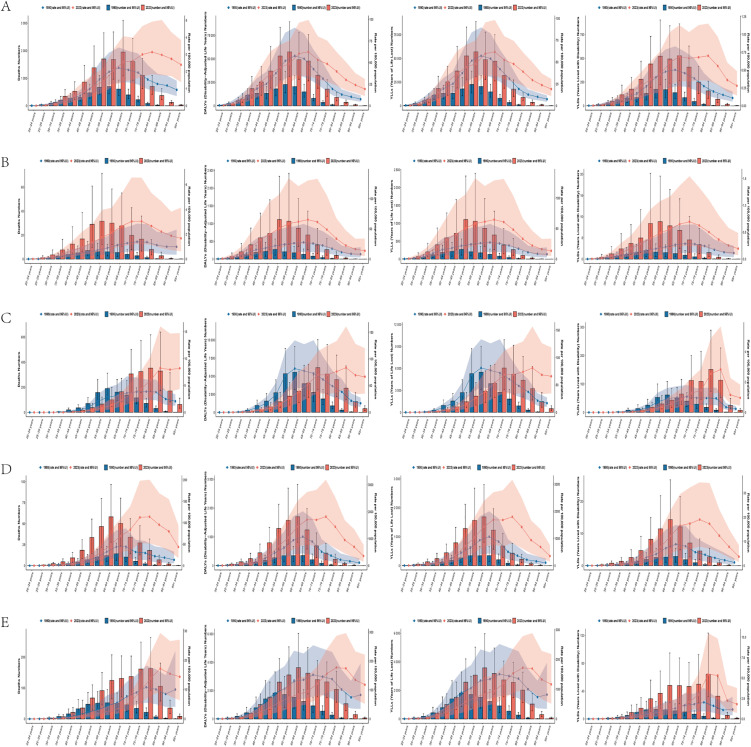
Trends from 1990 to 2023 by age group in number and age-standardized rates for LC-MR in five East Asian countries. (A) China; (B) Democratic People’s Republic of Korea; (C) Japan; (D) Mongolia; (E) Republic of Korea.

In 2023, the peak age groups for the highest disease burden varied, with 85–89 years in Japan and Republic of Korea, 65–79 years in Democratic People’s Republic of Korea, 75–84 years in Mongolia, and 80–84 years in China (Fig 3). From 1990 to 2023, the peak ages for age-standardized mortality and DALYs showed distinct shifts. In Japan, the peak age shifted from 80–84 and 60–64 years to 85–89 years, in Republic of Korea, from 70–84–80–89 years, and in Mongolia, from 65–74–75–84 years. The peak age in Democratic People’s Republic of Korea remained stable at 65–79 years. In China, the mortality peak shifted from 60–69–80–84 years, while the DALYs peak moved to 65–69 years, indicating a clear transition from an early-onset to a later-onset pattern (Fig 4).

### Burden of LC-MR by SDI

**Fig 5:**
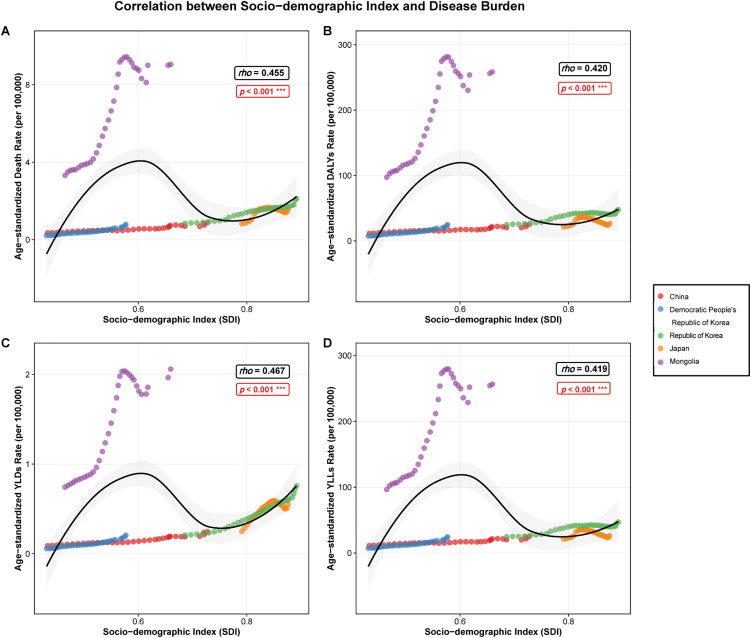
SDI correlation analysis in five East Asian countries. (A) Age-standardized Death Rate; (B) Age-standardized DALYs Rate; (C) Age-standardized YLDs Rate; (D) Age-standardized YLLs Rate.

The SDI exhibited a positive correlation with the rates of mortality, DALYs, YLDs, and YLLs for LC-MR across the five East Asian countries, with Pearson correlation coefficients (*rho*) of 0.455, 0.420, 0.467, and 0.419, respectively. Generally, the disease burden increased with higher SDI levels, with the notable exception of Mongolia, which showed a declining trend around 2010 (Fig 5).

### Decomposition analysis of changes in the disease burden of LC-MR

**Fig 6:**
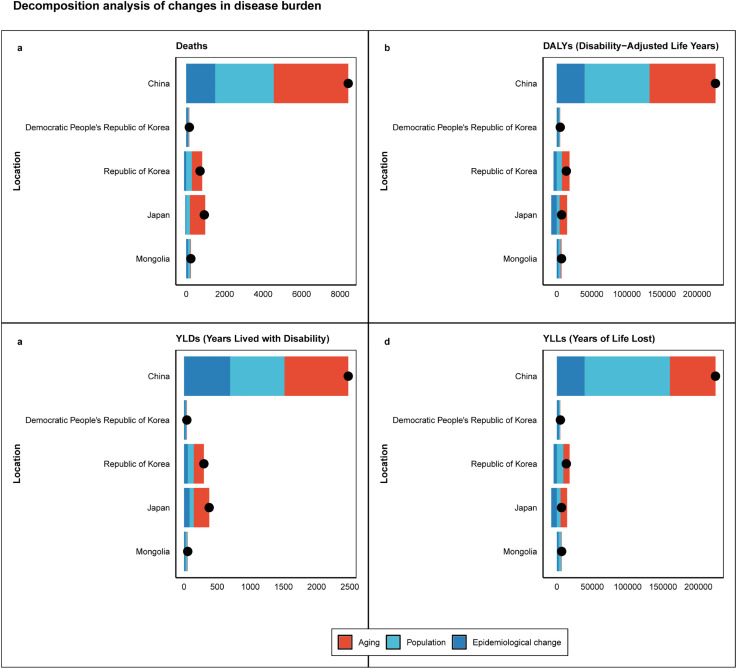
Decomposition analysis in five East Asian countries. (A) Deaths; (B) DALYs; (C) YLDs; (D) YLLs.

Decomposition analysis revealed significant country-specific differences in the drivers of changes in the disease burden of LC-MR across the five East Asian countries from 1990 to 2023 (Fig 6). In Japan and Republic of Korea, population ageing was the primary driver of increased disease burden. It contributed 84.36% and 153.54% to the increases in mortality and DALY rates in Japan, and 74.99% and 79.49% to those in Republic of Korea, respectively. In contrast, epidemiological changes reduced mortality and DALY rates by 4.84% and 111.64% in Japan, and by 15.59% and 33.68% in Republic of Korea (Table 2).

**Table 2:** A decomposition analysis in five East Asian countries from 1990 to 2023.

indicator type	Location	Overll difference	Aging	Population	Epidemiological change
Deaths	China	8421.83	3872.74 (45.98%)	3044.35 (36.15%)	1504.74 (17.87%)
	Japan	939.79	792.76 (84.36%)	192.49 (20.48%)	−45.46 (−4.84%)
	Democratic People’s Republic of Korea	162.1	28.98 (17.88%)	46.6 (28.75%)	86.51 (53.37%)
	Mongolia	241.36	30.44 (12.61%)	115.32 (47.78%)	95.6 (39.61%)
	Republic of Korea	720.69	540.43 (74.99%)	292.64 (40.61%)	−112.38 (−15.59%)
YLDs	Mongolia	55.26	8.01 (14.49%)	26.05 (47.15%)	21.2 (38.37%)
	Democratic People’s Republic of Korea	42.66	6.79 (15.91%)	12.28 (28.78%)	23.59 (55.31%)
	China	2476.48	962.75 (38.88%)	819.04 (33.07%)	694.7 (28.05%)
	Japan	379.08	232.9 (61.44%)	63.59 (16.77%)	82.59 (21.79%)
	Republic of Korea	299.23	153.18 (51.19%)	88.58 (29.6%)	57.47 (19.21%)
YLLs	Japan	6563.18	9217.49 (140.44%)	5213.77 (79.44%)	−7868.08 (−119.88%)
	China	224124.25	64359.03 (28.72%)	120584.14 (53.8%)	39181.07 (17.48%)
	Mongolia	6783.66	635.97 (9.38%)	3767.29 (55.53%)	2380.4 (35.09%)
	Democratic People’s Republic of Korea	4950.63	420.08 (8.49%)	1745.55 (35.26%)	2785 (56.26%)
	Republic of Korea	13359.89	8964.8 (67.1%)	9064.84 (67.85%)	−4669.75 (−34.95%)
DALYs	China	226296.28	93981.93 (41.53%)	92544.68 (40.9%)	39769.67 (17.57%)
	Mongolia	6844.44	1158.85 (16.93%)	3305.67 (48.3%)	2379.92 (34.77%)
	Democratic People’s Republic of Korea	5001.97	683.22 (13.66%)	1510.4 (30.2%)	2808.35 (56.14%)
	Republic of Korea	13656.62	10855.8 (79.49%)	7400.77 (54.19%)	−4599.95 (−33.68%)
	Japan	6939.55	10654.72 (153.54%)	4032.06 (58.1%)	−7747.23 (−111.64%)

In Democratic People’s Republic of Korea and Mongolia, epidemiological shifts were the primary driver of the increased disease burden, accounting for 53.37% and 39.61% of the rise in mortality, respectively. Population growth played a particularly prominent role in Mongolia, accounting for 47.78% and 48.30% of the increases in mortality and DALY rates. In China, all three factors jointly drove the growth in disease burden. The contributions of ageing, population growth, and epidemiological changes to mortality were 45.98%, 36.15%, and 17.87%, respectively, and to DALY rates were 41.53%, 40.90%, and 17.57%, respectively (Table 2).

### Prediction of the disease burden of LC-MR, 2023–2053

**Fig 7:**
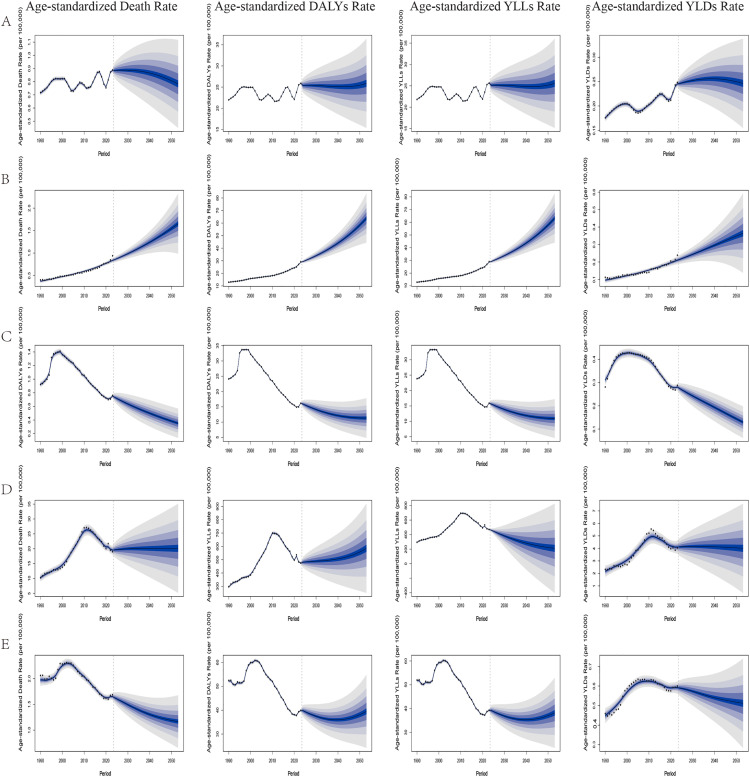
Bayesian age-period-cohort prediction of age-standardized rate. (A) China; (B) Democratic People’s Republic of Korea; (C) Japan; (D) Mongolia; (E) Republic of Korea.

Based on GBD 2023 data, we projected the future burden of LC-MR in the five East Asian countries to 2053 (Fig 7). The projections reveal a further divergence in disease burden patterns. Both Japan and Republic of Korea are projected to show a decline in age-standardized mortality rates, which are expected to fall to 0.35 and 1.17 per 100,000 person-years, respectively (Fig 7C and 7E). In contrast, Mongolia’s age-standardized DALY rate is projected to increase to 585.38 per 100,000 person-years and remain high. Democratic People’s Republic of Korea may experience the most acute deterioration, with the age-standardized mortality and DALY rates potentially reaching 1.65 and 63.94 per 100,000 person-years by 2053, representing increases of 96% and 122% from 2023 levels, respectively (Fig 7B and 7D). In contrast, all indicators for China are projected to remain relatively stable (Fig 7A).

## Discussion

Globally, the disease burden of liver cancer is undergoing a marked epidemiological transition from infectious to metabolic causes, posing an increasingly pressing public health challenge. Based on the GBD 2023 database, this study systematically evaluated the disease burden of LC-MR and driving factors in five East Asian countries, including China, Japan, Republic of Korea, Democratic People’s Republic of Korea, and Mongolia, from 1990 to 2023, and projected trends through 2053. The findings reveal significant heterogeneity across countries, sex disparities, and shifts in age structure within the disease burden of the region. These patterns are primarily driven by the synergistic effects of population aging, epidemiological changes, and unequal socioeconomic development.

However, given the biological mechanisms underlying traditional etiologies of liver cancer, priority should be given to the biological synergy between metabolic risk factors and conventional liver disease causes, such as viral hepatitis and alcohol. As reviewed by Li et al., MASLD and chronic hepatitis B may exacerbate liver fibrosis and hepatocellular carcinoma through synergistic effects [16]. A review by Imran Hasanoglu et al. further noted that when MASLD coexists with viral hepatitis including hepatitis B and C, it may exacerbate liver lesions through additive or synergistic effects, leading to complications such as cirrhosis, liver failure and hepatocellular carcinoma [17]. Additionally, a review by Gao et al. found that alcohol and metabolic risk factors can act synergistically to accelerate the development of steatohepatitis, liver fibrosis and hepatocellular carcinoma [18]. These mechanisms imply that in East Asian countries where chronic hepatitis B remains prevalent and alcohol consumption is not negligible, particularly China and Mongolia, the additive or multiplicative effects of metabolic, infectious, and alcoholic risks may further amplify liver cancer burden.

Overall, the five East Asian countries showed clear distinct trends in the disease burden of LC-MR, each of which was driven by a unique underlying process. The liver cancer disease burden of Mongolia attributed to metabolic risks continuously ranked highest from 1990 to 2023. It showed an overall growing trend with an increase followed by a reduction, peaking in 2010. With the exception of a little increase in the period it lived with the disease rate, Republic of Korea, which consistently ranked second, displayed an overall declining trend. Democratic People’s Republic of Korea showed an overall rising trend, with its disease burden moving from the lowest to the third, in contrast to the persistent declining trend of Japan. Additionally, China consistently ranked fourth, maintaining a stable state of persistent burden.

Between 1990 and 2023, Republic of Korea and Japan, two nations with high SDI scores, both showed decreasing trends in DALY and age-standardized mortality rates. This accomplishment is primarily due to the multifunctional, comprehensive prevention and control strategies put into effect by both countries, which include important efforts like long-term monitoring for those at high risk of MASLD and early screening systems supported by general health insurance. Japan has concentrated on NAFLD, or non-alcoholic fatty liver disease, as a major risk factor. Major risk factors for the development of liver cancer have been found by long-term monitoring of patients [19]. Furthermore, Japan implements regular liver function and radiological monitoring, significantly improving the detection rate of early-stage liver cancer and improving the prognosis of patients [20]. Similar to Japan, Republic of Korea’s public health insurance system provides institutional support for early liver cancer diagnosis and treatment, ensuring accessibility to screening and clinical care. Studies indicated that despite rising NAFLD prevalence, its 10-year progression rate to liver cancer remains only 0.77% [21]. The rapidly aging populations have become the primary drivers of changing disease burdens for LC-MR in both Japan and Republic of Korea. A prospective cohort study in Japan confirmed that metabolic-related diseases prevalent in the elderly population, such as obesity and hyperglycemia, significantly increase the risk of HCC [22]. Moreover, the average age of newly diagnosed patients has shown an upward trend in recent years [23]. Korean cohort studies similarly reveal a marked increase in HCC incidence among patients aged 80 years and older [24,25]. This trend suggests that future prevention and control efforts in both countries should shift toward refined screening and stratified management targeting elderly high-risk populations.

In contrast, the disease burden of LC-MR in Mongolia and Democratic People’s Republic of Korea has shown a continually rising trend, mainly driven by serious epidemiological changes. Notably, the contribution of metabolic risk factors in Mongolia may be amplified through their interaction with traditional liver cancer etiologies, particularly chronic HBV/HCV infection and alcohol-related liver disease, both of which remain highly prevalent in the country. In terms of metabolic risk attribution, distinctive dietary habits in Mongolia and the highest global rates of obesity and hyperglycemia are directly related to high disease burden in the country [26]. Following socioeconomic and lifestyle changes, Mongolia has developed a diet low in dietary fiber and high in fat and salt. 49.4% of adults are overweight or obese. The severe trend in disease burden is caused by both this dietary pattern and increasing hyperglycemia levels. Meanwhile, a study showed a tendency for metabolic reprogramming in the molecular features of liver cancer in Mongolia. Amino acid biosynthesis pathways exhibit abnormal activation, and around 30% of differently expressed genes take part in metabolic reprogramming processes. Important genes, including UPP2 and PCK1 have significant expression alterations [27], providing molecular-level evidence that metabolic disorders are the main cause of this disease. Furthermore, the cumulative effects of multiple metabolic abnormalities further amplify carcinogenic risks [28]. The carcinogenic potential of individual metabolic components is greatly increased when metabolic syndrome components like diabetes and hypertension are combined. However, Democratic People’s Republic of Korea has a relatively underdeveloped public health system, lacking early screening and chronic disease management. Relevant immigration statistics indicate a high prevalence of metabolic syndrome and weight gain, both of which increase disease burden [29,30]. Notably, disease burden in Mongolia has been decreasing since a national improved prevention and control strategy was implemented in 2010 [26], indicating that active and systematic health policies may successfully curb the adverse trend of disease burden.

Population growth, population aging, and epidemiological changes are the three main factors contributing to the disease burden of LC-MR in China. Aging increases the risk of LC-MR, and the large population base provides a potential host for the occurrence of the disease. The cumulative effects of LC-MR-related damage in the elderly significantly raise the risk of HCC, and research by Tang et al. confirms that population aging increases the prevalence of metabolic diseases [31]. This suggests that the lagged effects of metabolic factors in the elderly population cannot be ignored. Additionally, the prevalence of metabolic risk factors such as diabetes and obesity has become a significant factor in the increased incidence of HCC, coupled with changes in lifestyle and socioeconomic development [32]. Among these countries, the annual average increase in the prevalence of non-alcoholic fatty liver disease (NAFLD) in China (AAPC = 1.30) is much higher than the global average (AAPC = 0.91). This difference is directly related to the ongoing increase in the incidence of metabolic syndrome, diabetes, and obesity in the country [33,34]. Rapid urbanization, unhealthy dietary and lifestyle habits, and genetic susceptibility are some factors contributing to the higher prevalence of NAFLD. The long-term role of metabolic risk factors in liver disease progression was confirmed by a large-scale cohort study from the China Kadoorie Biobank—a major prospective cohort in China [32]—which clearly demonstrated significant associations between obesity, diabetes, abnormal blood glucose levels, and severe liver diseases, including liver cancer. This finding is further confirmed by several additional studies. For example, by regulating cellular gene expression, high fasting blood glucose levels can abnormally increase the risk of liver cancer [35]. One of the main risk factors for liver cancer in China is the significant rise in the incidence of high BMI from 1990 to 2021 [11]. Due to multiple factors, China ranks among the highest globally in absolute cases of LC-MR. Although the age-standardized incidence rate has remained stable, the overall situation continues to face sustained pressure. Furthermore, considering the high burden of chronic hepatitis B in China, the synergistic interaction between metabolic risk factors and HBV may contribute to the persistently high absolute cases of LC-MR, warranting integrated prevention strategies targeting both etiologies.

Inconsistent with research by Harriet, Sungchul, and others [36,37], our study demonstrates that the disease burden of LC-MR is often higher among males than females in East Asia. The difference results from a combination of biological mechanisms, behavioral patterns, and social environments. Behavioral risk factors such as higher smoking and alcohol consumption rates among males synergize with metabolic risks [38]. Additionally, the protective effects of estrogen on metabolic regulation and anti-inflammatory processes in females partially reduce their disease risk [39]. However, there is considerable variation in sex disparities and contributing variables between countries, which reflects the impact of healthcare accessibility and socioeconomic development levels. The greatest sex disparity is observed in Republic of Korea, which may be related to poor health management among males and increased exposure to hazardous behaviors. A nationwide cohort study confirmed that sex disparities in metabolism-related HCC are primarily driven by the high prevalence of high-risk behavioral patterns such as smoking and alcohol consumption among males [40]. Disease burden patterns across sexes show a distinct sex paradox in China. While the age-standardized burden of disease is still increasing for males, it is trending decrease for females. This finding diverges from the general rising trend in the burden of LC-MR observed by Tang et al., where the increase was greater in males, and requires further evidence [31]. In particular, the prevalence of liver cancer disease declined in both Democratic People’s Republic of Korea and Mongolia, with the burden rising far more quickly in females than in males. This brings a challenge to the traditional view that liver cancer mostly affects males. Inadequate medical responses and greater exposure of females to metabolic risks during socioeconomic shifts may be related to this concern. According to US research, in a situation of metabolic disorders, female sex may be an independent risk factor for liver cancer [41]. LC-MR currently affects more females than males, with a greater prevalence and faster growing rate, according to research found in Mongolia [26]. These differences show that the sex distribution of LC-MR in East Asia is impacted by social changes, risk exposures, and healthcare capacities among nations in addition to biological and behavioral variables. This necessitates the implementation of specific by sex prevention and control strategies.

Between 1990 and 2023, the age distribution of LC-MR in five East Asian countries showed a trend toward older age groups. The peak age of onset for LC‑MR has been progressively delayed in Japan, Republic of Korea, Mongolia, and China. In high SDI countries such as Japan, the peak age for the age‑standardized DALYs rate of LC‑MR shifted from 60–64 years to 85–89 years, a change that may be associated with the rising proportion of elderly populations, long‑term accumulation of metabolic disorders, and the relatively well‑established systems for disease diagnosis and registration [42]. In China, the peak age for age-standardized mortality rates shifted from 60–69 years to 80–84 years. This change aligns with the population aging process and may also reflect the long-term shift in the etiological spectrum of liver cancer from viral hepatitis toward metabolic risk factors [43]. In contrast, low SDI countries such as Democratic People’s Republic of Korea showed minimal changes in the age-specific distribution, suggesting they remain at a different stage of epidemiological transition [44]. Overall, changes in age distribution and disease burden exhibit significant cross-country heterogeneity, closely related to differences in population structure and socioeconomic development levels.

This study investigated the overall distribution and trends of LC-MR from 1990 to 2023. We performed subgroup analyses based on age and sex across different countries to evaluate the independent effects of socioeconomic development, demographic shifts, and epidemiological factors, and predicted changes in disease burden from 2023 to 2053. But our research also has some limitations. First, the estimates in this study were derived from the GBD database, where the level of disease burden relies on the integration of multiple data sources and modeling inferences. Differences in disease reporting systems and data collection methods among countries may affect the accuracy of the results, especially in Democratic People’s Republic of Korea and Mongolia. Second, the GBD database lacks detailed statistics on specific metabolic risk factors, such as high fasting plasma glucose and obesity. Finally, the predictions were mainly based on past data and did not fully include possible future changes such as new public health measures, better access to healthcare, or shifts in society. In the future, each country should improve early detection and treatment of metabolic liver diseases according to its own health situation. At the same time, it is important to start long-term studies across multiple hospitals in East Asia to accurately measure how specific metabolic risk factors contribute to the disease.

The results of this study show that the burden of LC-MR in East Asia has continued to rise over the past 30 years, with distinct variation patterns across countries that are associated with sex, population age structure, shifts in disease spectrum, and levels of socioeconomic development. East Asian countries should collaborate through joint prevention and control measures and implement targeted interventions for high- risk populations.

## Conclusion

The disease burden of LC-MR in East Asia shows significant across-country heterogeneity, driven mainly by population ageing and the prevalence of metabolic risks. Differentiated public health strategies are needed. It is essential to strengthen interventions for metabolic risks, enhance early screening, and implement precise prevention for high-risk populations to reduce the regional burden of liver cancer.
